# Supplementary material for: Severe Acute Respiratory Syndrome Coronavirus 2 Variants of Concern: A Perspective for Emerging More Transmissible and Vaccine-Resistant Strains
Source: Viruses. 2022 Apr 16;14(4):827. doi: 10.3390/v14040827 (PMC9029021; doi:10.3390/v14040827)
Supplement: Supplementary file 1 [file viruses-14-00827-s001.zip › viruses-1621994-supplementary.pdf]

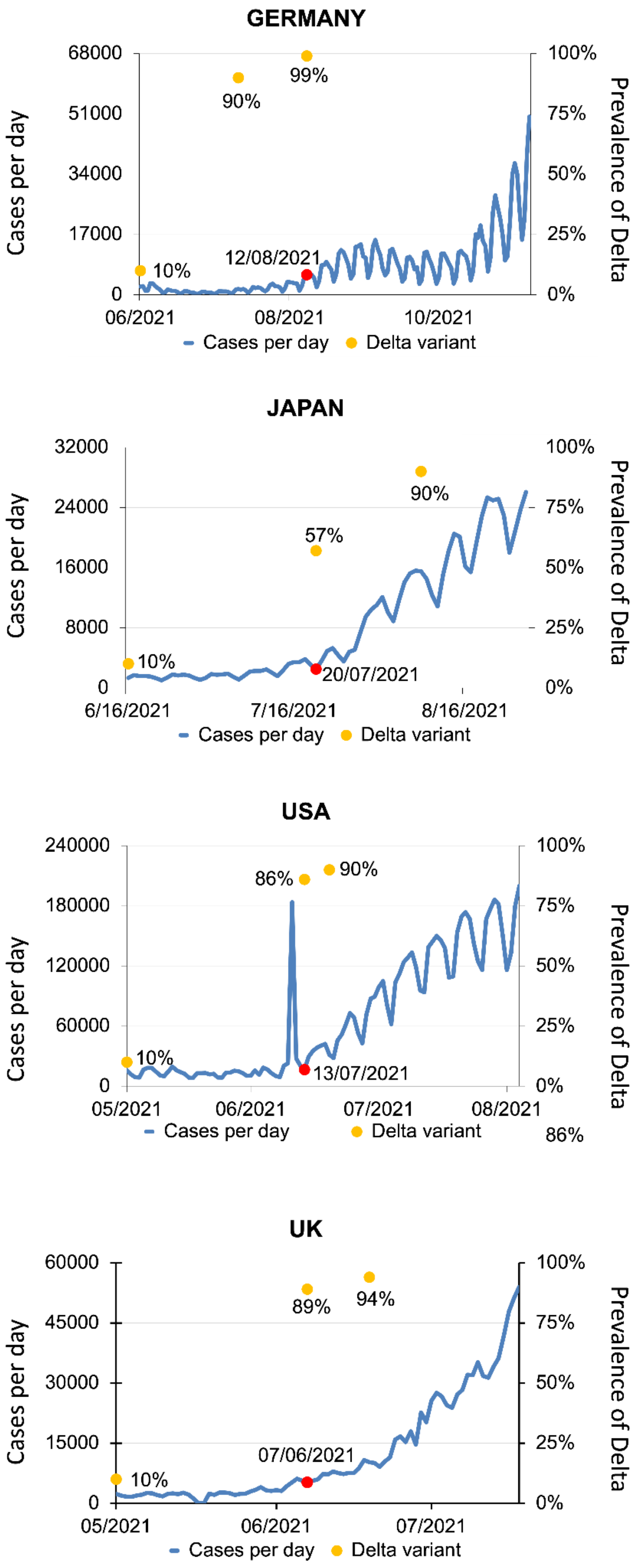

Figure S1. Number of COVID-19 cases and the prevalence of the SARS-CoV-2 Delta variant in the UK, Japan, USA, and Germany. Graphs of the number of cases (blue line) and the percentage of the Delta variant (orange circles) as a function of time. Red circles show the beginning of a new COVID-19 peak case. All data were obtained from World in Data, WHO, CDC, UK Gov and Outbreak [1,148–151].

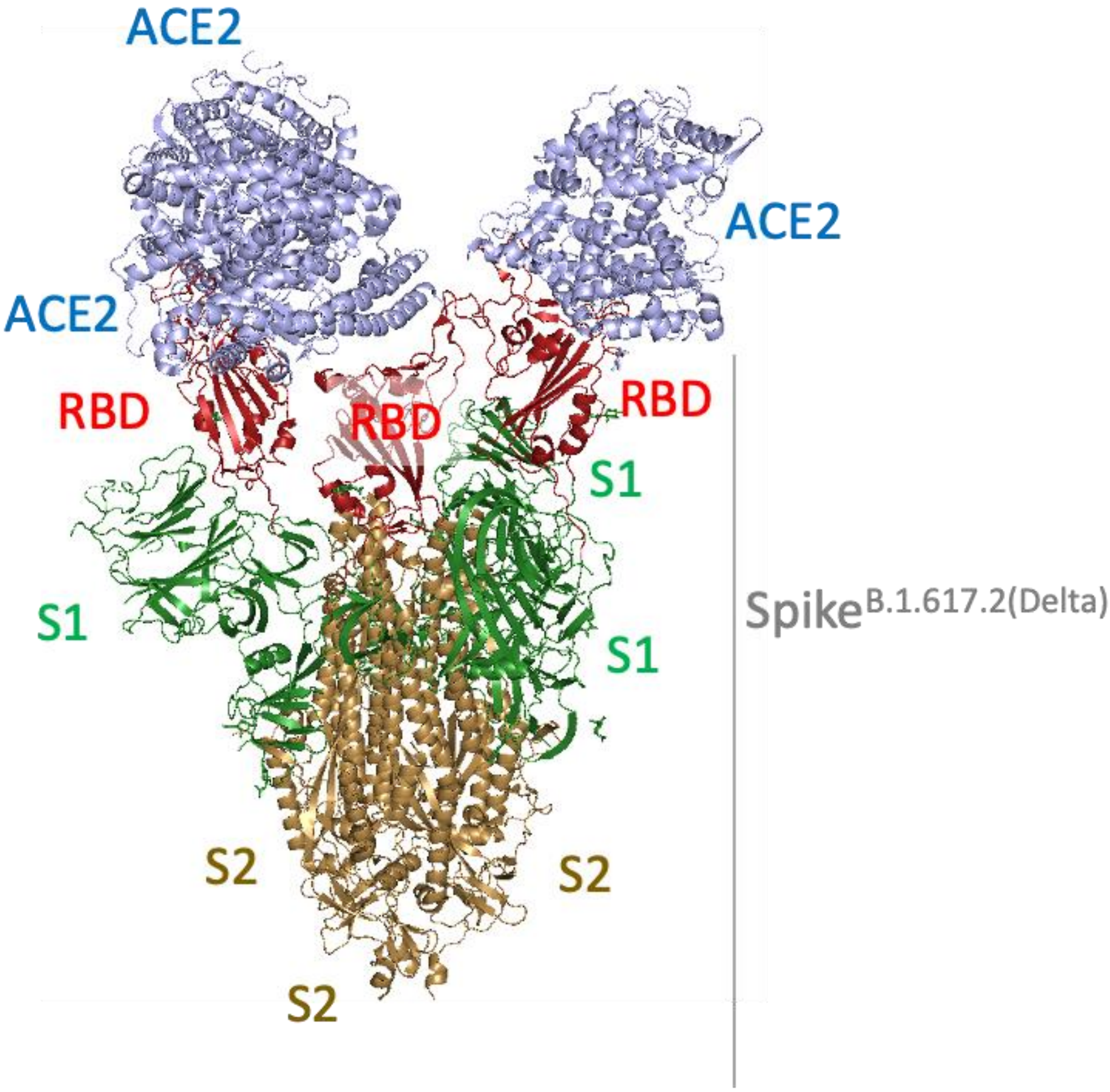

Figure S2. Cryo-EM structure of SARS-CoV-2 Spike$^{B.1.617.2(Delta)}$ in complex with Angiotensin-converting enzyme 2 (ACE2) ectodomain. ACE2 is colored in blue, Spike protein structure in red (RBD domain), green (S1 subunit not considering the RBD domain) and brown (S2 domain) (protein data bank, PDB, 7V8A, not published).

A

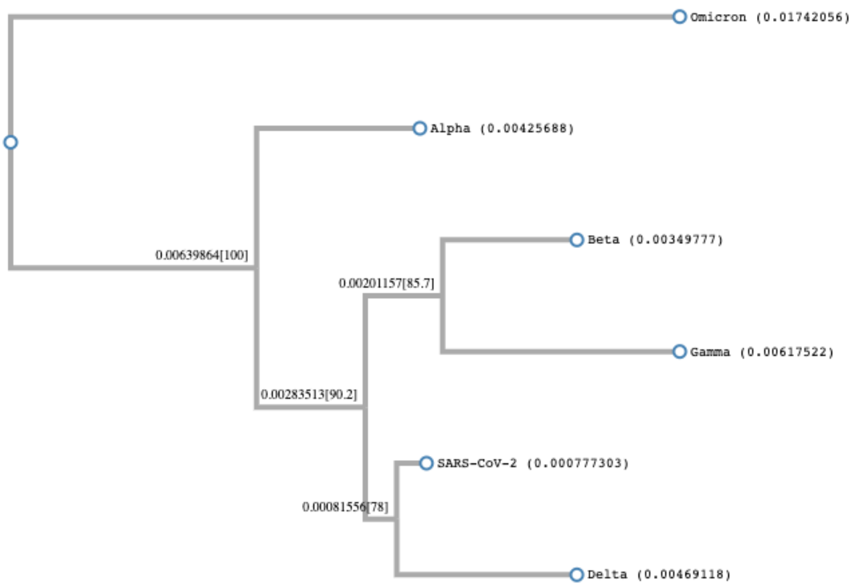

B

```
SARS-CoV-2          MFVFLVLLPLVSSQCVNLTTRTQLPPAYTNSFTRGVYYPDKVFRSSVLHSTQDLFLPFFS   60
Delta/B.1.617.2     MFVFLVLLPLVSSQCVNLRTRTQLPPAYTNSFTRGVYYPDKVFRSSVLHSTQDLFLPFFS   60
Omicron/B.1.1.529   MFVFLVLLPLVSSQCVNLTTRTQLPPAYTNSFTRGVYYPDKVFRSSVLHSTQDLFLPFFS   60
Alpha/B.1.1.7       MFVFLVLLPLVSSQCVNLTTRTQLPPAYTNSFTRGVYYPDKVFRSSVLHSTQDLFLPFFS   60
P.1/Gamma           MFVFLVLLPLVSSQCVNFTNRTQLPSAYTNSFTRGVYYPDKVFRSSVLHSTQDLFLPFFS   60
Beta/B.1.351        MFVFLVLLPLVSSQCVNFTTRTQLPPAYTNSFTRGVYYPDKVFRSSVLHSTQDLFLPFFS   60
                    *****************: .***** **********************************

SARS-CoV-2          NVTWFHAIHVSGTNGTKRFDNPVLPFNDGVYFASTEKSNIIRGWIFGTTLDSKTQSLLIV   120
Delta/B.1.617.2     NVTWFHAIHVSGTNGTKRFDNPVLPFNDGVYFASTEKSNIIRGWIFGTTLDSKTQSLLIV   120
Omicron/B.1.1.529   NVTWFHVI--SGTNGTKRFDNPVLPFNDGVYFASIEKSNIIRGWIFGTTLDSKTQSLLIV   118
Alpha/B.1.1.7       NVTWFHAI--SGTNGTKRFDNPVLPFNDGVYFASTEKSNIIRGWIFGTTLDSKTQSLLIV   118
P.1/Gamma           NVTWFHAIHVSGTNGTKRFDNPVLPFNDGVYFASTEKSNIIRGWIFGTTLDSKTQSLLIV   120
Beta/B.1.351        NVTWFHAIHVSGTNGTKRFANPVLPFNDGVYFASTEKSNIIRGWIFGTTLDSKTQSLLIV   120
                    ******.*  ********* **************.*************************

SARS-CoV-2          NNATNVVIKVCEFQFCNDPFLGVYYHKNNKSWMESEFRVYSSANNCTFEYVSQPFLMDLE   180
Delta/B.1.617.2     NNATNVVIKVCEFQFCNDPFLDVYYHKNNKSWMESG--VYSSANNCTFEYVSQPFLMDLE   180
Omicron/B.1.1.529   NNATNVVIKVCEFQFCNDPFLD---HKNNKSWMESEFRVYSSANNCTFEYVSQPFLMDLE   175
Alpha/B.1.1.7       NNATNVVIKVCEFQFCNDPFLGVY-HKNNKSWMESEFRVYSSANNCTFEYVSQPFLMDLE   177
P.1/Gamma           NNATNVVIKVCEFQFCNYPFLGVYYHKNNKSWMESEFRVYSSANNCTFEYVSQPFLMDLE   180
Beta/B.1.351        NNATNVVIKVCEFQFCNDPFLGVYYHKNNKSWMESEFRVYSSANNCTFEYVSQPFLMDLE   180
                    ***************** ***.   **********   **********************

SARS-CoV-2          GKQGNFKNLREFVFKNIDGYFKIYSKHTPINL--VRDLPQGFSALEPLVDLPIGINITRF   238
Delta/B.1.617.2     GKQGNFKNLREFVFKNIDGYFKIYSKHTPINL--VRDLPQGFSALEPLVDLPIGINITRF   238
Omicron/B.1.1.529   GKQGNFKNLREFVFKNIDGYFKIYSKHTPIIVREPEDLPQGFSALEPLVDLPIGINITRF   235
Alpha/B.1.1.7       GKQGNFKNLREFVFKNIDGYFKIYSKHTPINL--VRDLPQGFSALEPLVDLPIGINITRF   235
P.1/Gamma           GKQGNFKNLSEFVFKNIDGYFKIYSKHTPINL--VRDLPQGFSALEPLVDLPIGINITRF   238
Beta/B.1.351        GKQGNFKNLREFVFKNIDGYFKIYSKHTPINL--VRGLPQGFSALEPLVDLPIGINITRF   238
                    ********* ******************** :   ..***********************
```

```
SARS-CoV-2          QTLLALHRSYLTPGDSSSGWTAGAAAYYVGYLQPRTFLLKYNENGTITDAVDCALDPLSE   298
Delta/B.1.617.2     QTLLALHRSYLTPGDSSSGWTAGAAAYYVGYLQPRTFLLKYNENGTITDAVDCALDPLSE   298
Omicron/B.1.1.529   QTLLALHRSYLTPGDSSSGWTAGAAAYYVGYLQPRTFLLKYNENGTITDAVDCALDPLSE   295
Alpha/B.1.1.7       QTLLALHRSYLTPGDSSSGWTAGAAAYYVGYLQPRTFLLKYNENGTITDAVDCALDPLSE   295
P.1/Gamma           QTLLALHRSYLTPGDSSSGWTAGAAAYYVGYLQPRTFLLKYNENGTITDAVDCALDPLSE   298
Beta/B.1.351        QT---LHRSYLTPGDSSSGWTAGAAAYYVGYLQPRTFLLKYNENGTITDAVDCALDPLSE   295
                    **   *******************************************************

SARS-CoV-2          TKCTLKSFTVEKGIYQTSNFRVQPTESIVRFPNITNLCPFGEVFNATRFASVYAWNRKRI   358
Delta/B.1.617.2     TKCTLKSFTVEKGIYQTSNFRVQPTESIVRFPNITNLCPFGEVFNATRFASVYAWNRKRI   358
Omicron/B.1.1.529   TKCTLKSFTVEKGIYQTSNFRVQPTESIVRFPNITNLCPFDEVFNATRFASVYAWNRKRI   355
Alpha/B.1.1.7       TKCTLKSFTVEKGIYQTSNFRVQPTESIVRFPNITNLCPFGEVFNATRFASVYAWNRKRI   355
P.1/Gamma           TKCTLKSFTVEKGIYQTSNFRVQPTESIVRFPNITNLCPFGEVFNATRFASVYAWNRKRI   358
Beta/B.1.351        TKCTLKSFTVEKGIYQTSNFRVQPTESIVRFPNITNLCPFGEVFNATRFASVYAWNRKRI   355
                    ****************************************.*******************

SARS-CoV-2          SNCVADYSVLYNSASFSTFKCYGVSPTKLNDLCFTNVYADSFVIRGDEVRQIAPGQTGKI   418
Delta/B.1.617.2     SNCVADYSVLYNSASFSTFKCYGVSPTKLNDLCFTNVYADSFVIRGDEVRQIAPGQTGKI   418
Omicron/B.1.1.529   SNCVADYSVLYNLAPFFTFKCYGVSPTKLNDLCFTNVYADSFVIRGDEVRQIAPGQTGNI   415
Alpha/B.1.1.7       SNCVADYSVLYNSASFSTFKCYGVSPTKLNDLCFTNVYADSFVIRGDEVRQIAPGQTGKI   415
P.1/Gamma           SNCVADYSVLYNSASFSTFKCYGVSPTKLNDLCFTNVYADSFVIRGDEVRQIAPGQTGTI   418
Beta/B.1.351        SNCVADYSVLYNSASFSTFKCYGVSPTKLNDLCFTNVYADSFVIRGDEVRQIAPGQTGNI   415
                    ************ * * *****************************************.*

SARS-CoV-2          ADYNYKLPDDFTGCVIAWNSNNLDSKVGGNYNYLYRLFRKSNLKPFERDISTEIYQAGST   478
Delta/B.1.617.2     ADYNYKLPDDFTGCVIAWNSNNLDSKVGGNYNYRYRLFRKSNLKPFERDISTEIYQAGSK   478
Omicron/B.1.1.529   ADYNYKLPDDFTGCVIAWNSNKLDSKVSGNYNYLYRLFRKSNLKPFERDISTEIYQAGNK   475
Alpha/B.1.1.7       ADYNYKLPDDFTGCVIAWNSNNLDSKVGGNYNYLYRLFRKSNLKPFERDISTEIYQAGST   475
P.1/Gamma           ADYNYKLPDDFTGCVIAWNSNNLDSKVGGNYNYLYRLFRKSNLKPFERDISTEIYQAGST   478
Beta/B.1.351        ADYNYKLPDDFTGCVIAWNSNNLDSKVGGNYNYLYRLFRKSNLKPFERDISTEIYQAGST   475
                    *********************:*****.***** ************************..

                                                ⬇
SARS-CoV-2          PCNGVEGFNCYFPLQSYGFQPTNGVGYQPYRVVVLSFELLHAPATVCGPKKSTNLVKNKC   538
Delta/B.1.617.2     PCNGVEGFNCYFPLQSYGFQPTNGVGYQPYRVVVLSFELLHAPATVCGPKKSTNLVKNKC   538
Omicron/B.1.1.529   PCNGVAGFNCYFPLRSYSFRPTYGVGHQPYRVVVLSFELLHAPATVCGPKKSTNLVKNKC   535
Alpha/B.1.1.7       PCNGVEGFNCYFPLQSYGFQPTYGVGYQPYRVVVLSFELLHAPATVCGPKKSTNLVKNKC   535
P.1/Gamma           PCNGVKGFNCYFPLQSYGFQPTYGVGYQPYRVVVLSFELLHAPATVCGPKKSTNLVKNKC   538
Beta/B.1.351        PCNGVKGFNCYFPLQSYGFQPTYGVGYQPYRVVVLSFELLHAPATVCGPKKSTNLVKNKC   535
                    ***** ********:**.*:** ***:*********************************

SARS-CoV-2          VNFNFNGLTGTGVLTESNKKFLPFQQFGRDIADTTDAVRDPQTLEILDITPCSFGGVSVI   598
Delta/B.1.617.2     VNFNFNGLTGTGVLTESNKKFLPFQQFGRDIADTTDAVRDPQTLEILDITPCSFGGVSVI   598
Omicron/B.1.1.529   VNFNFNGLKGTGVLTESNKKFLPFQQFGRDIADTTDAVRDPQTLEILDITPCSFGGVSVI   595
Alpha/B.1.1.7       VNFNFNGLTGTGVLTESNKKFLPFQQFGRDIDDTTDAVRDPQTLEILDITPCSFGGVSVI   595
P.1/Gamma           VNFNFNGLTGTGVLTESNKKFLPFQQFGRDIADTTDAVRDPQTLEILDITPCSFGGVSVI   598
Beta/B.1.351        VNFNFNGLTGTGVLTESNKKFLPFQQFGRDIADTTDAVRDPQTLEILDITPCSFGGVSVI   595
                    ********.********************** ****************************
                                   ⬇
SARS-CoV-2          TPGTNTSNQVAVLYQDVNCTEVPVAIHADQLTPTWRVYSTGSNVFQTRAGCLIGAEHVNN   658
Delta/B.1.617.2     TPGTNTSNQVAVLYQGVNCTEVPVAIHADQLTPTWRVYSTGSNVFQTRAGCLIGAEHVNN   658
Omicron/B.1.1.529   TPGTNTSNQVAVLYQGVNCTEVPVAIHADQLTPTWRVYSTGSNVFQTRAGCLIGAEYVNN   655
Alpha/B.1.1.7       TPGTNTSNQVAVLYQGVNCTEVPVAIHADQLTPTWRVYSTGSNVFQTRAGCLIGAEHVNN   655
P.1/Gamma           TPGTNTSNQVAVLYQGVNCTEVPVAIHADQLTPTWRVYSTGSNVFQTRAGCLIGAEYVNN   658
Beta/B.1.351        TPGTNTSNQVAVLYQGVNCTEVPVAIHADQLTPTWRVYSTGSNVFQTRAGCLIGAEHVNN   655
                    ***************.****************************************:***

                                          S1/S2
                                            ▽
SARS-CoV-2          SYECDIPIGAGICASYQTQTNSPRRARSVASQSIIAYTMSLGAENSVAYSNNSIAIPTNF   718
Delta/B.1.617.2     SYECDIPIGAGICASYQTQTNSRRRARSVASQSIIAYTMSLGAENSVAYSNNSIAIPTNF   718
Omicron/B.1.1.529   SYECDIPIGAGICASYQTQTKSHRRARSVASQSIIAYTMSLGAENSVAYSNNSIAIPTNF   715
Alpha/B.1.1.7       SYECDIPIGAGICASYQTQTNSHRRARSVASQSIIAYTMSLGAENSVAYSNNSIAIPINF   715
P.1/Gamma           SYECDIPIGAGICASYQTQTNSPRRARSVASQSIIAYTMSLGAENSVAYSNNSIAIPTNF   718
Beta/B.1.351        SYECDIPIGAGICASYQTQTNSPRRARSVASQSIIAYTMSLGVENSVAYSNNSIAIPTNF   715
```

```
                                   ********************:* *******************.************** **

SARS-CoV-2            TISVTTEILPVSMTKTSVDCTMYICGDSTECSNLLLQYGSFCTQLNRALTGIAVEQDKNT    778
Delta/B.1.617.2       TISVTTEILPVSMTKTSVDCTMYICGDSTECSNLLLQYGSFCTQLNRALTGIAVEQDKNT    778
Omicron/B.1.1.529     TISVTTEILPVSMTKTSVDCTMYICGDSTECSNLLLQYGSFCTQLKRALTGIAVEQDKNT    775
Alpha/B.1.1.7         TISVTTEILPVSMTKTSVDCTMYICGDSTECSNLLLQYGSFCTQLNRALTGIAVEQDKNT    775
P.1/Gamma             TISVTTEILPVSMTKTSVDCTMYICGDSTECSNLLLQYGSFCTQLNRALTGIAVEQDKNT    778
Beta/B.1.351          TISVTTEILPVSMTKTSVDCTMYICGDSTECSNLLLQYGSFCTQLNRALTGIAVEQDKNT    775
                      *********************************************:**************

                                                          S2′
                                                          ▽
SARS-CoV-2            QEVFAQVKQIYKTPPIKDFGGFNFSQILPDPSKPSKRSFIEDLLFNKVTLADAGFIKQYG    838
Delta/B.1.617.2       QEVFAQVKQIYKTPPIKDFGGFNFSQILPDPSKPSKRSFIEDLLFNKVTLADAGFIKQYG    838
Omicron/B.1.1.529     QEVFAQVKQIYKTPPIKYFGGFNFSQILPDPSKPSKRSFIEDLLFNKVTLADAGFIKQYG    835
Alpha/B.1.1.7         QEVFAQVKQIYKTPPIKDFGGFNFSQILPDPSKPSKRSFIEDLLFNKVTLADAGFIKQYG    835
P.1/Gamma             QEVFAQVKQIYKTPPIKDFGGFNFSQILPDPSKPSKRSFIEDLLFNKVTLADAGFIKQYG    838
Beta/B.1.351          QEVFAQVKQIYKTPPIKDFGGFNFSQILPDPSKPSKRSFIEDLLFNKVTLADAGFIKQYG    835
                      ***************** ******************************************

SARS-CoV-2            DCLGDIAARDLICAQKFNGLTVLPPLLTDEMIAQYTSALLAGTITSGWTFGAGAALQIPF    898
Delta/B.1.617.2       DCLGDIAARDLICAQKFNGLTVLPPLLTDEMIAQYTSALLAGTITSGWTFGAGAALQIPF    898
Omicron/B.1.1.529     DCLGDIAARDLICAQKFKGLTVLPPLLTDEMIAQYTSALLAGTITSGWTFGAGAALQIPF    895
Alpha/B.1.1.7         DCLGDIAARDLICAQKFNGLTVLPPLLTDEMIAQYTSALLAGTITSGWTFGAGAALQIPF    895
P.1/Gamma             DCLGDIAARDLICAQKFNGLTVLPPLLTDEMIAQYTSALLAGTITSGWTFGAGAALQIPF    898
Beta/B.1.351          DCLGDIAARDLICAQKFNGLTVLPPLLTDEMIAQYTSALLAGTITSGWTFGAGAALQIPF    895
                      *****************:******************************************

SARS-CoV-2            AMQMAYRFNGIGVTQNVLYENQKLIANQFNSAIGKIQDSLSSTASALGKLQDVVNQNAQA    958
Delta/B.1.617.2       AMQMAYRFNGIGVTQNVLYENQKLIANQFNSAIGKIQDSLSSTASALGKLQNVVNQNAQA    958
Omicron/B.1.1.529     AMQMAYRFNGIGVTQNVLYENQKLIANQFNSAIGKIQDSLSSTASALGKLQDVVNHNAQA    955
Alpha/B.1.1.7         AMQMAYRFNGIGVTQNVLYENQKLIANQFNSAIGKIQDSLSSTASALGKLQDVVNQNAQA    955
P.1/Gamma             AMQMAYRFNGIGVTQNVLYENQKLIANQFNSAIGKIQDSLSSTASALGKLQDVVNQNAQA    958
Beta/B.1.351          AMQMAYRFNGIGVTQNVLYENQKLIANQFNSAIGKIQDSLSSTASALGKLQDVVNQNAQA    955
                      ***************************************************:***:****

SARS-CoV-2            LNTLVKQLSSNFGAISSVLNDILSRLDKVEAEVQIDRLITGRLQSLQTYVTQQLIRAAEI    1018
Delta/B.1.617.2       LNTLVKQLSSNFGAISSVLNDILSRLDKVEAEVQIDRLITGRLQSLQTYVTQQLIRAAEI    1018
Omicron/B.1.1.529     LNTLVKQLSSKFGAISSVLNDIFSRLDKVEAEVQIDRLITGRLQSLQTYVTQQLIRAAEI    1015
Alpha/B.1.1.7         LNTLVKQLSSNFGAISSVLNDILARLDKVEAEVQIDRLITGRLQSLQTYVTQQLIRAAEI    1015
P.1/Gamma             LNTLVKQLSSNFGAISSVLNDILSRLDKVEAEVQIDRLITGRLQSLQTYVTQQLIRAAEI    1018
Beta/B.1.351          LNTLVKQLSSNFGAISSVLNDILSRLDKVEAEVQIDRLITGRLQSLQTYVTQQLIRAAEI    1015
                      **********:***********::************************************

SARS-CoV-2            RASANLAATKMSECVLGQSKRVDFCGKGYHLMSFPQSAPHGVVFLHVTYVPAQEKNFTTA    1078
Delta/B.1.617.2       RASANLAATKMSECVLGQSKRVDFCGKGYHLMSFPQSAPHGVVFLHVTYVPAQEKNFTTA    1078
Omicron/B.1.1.529     RASANLAATKMSECVLGQSKRVDFCGKGYHLMSFPQSAPHGVVFLHVTYVPAQEKNFTTA    1075
Alpha/B.1.1.7         RASANLAATKMSECVLGQSKRVDFCGKGYHLMSFPQSAPHGVVFLHVTYVPAQEKNFTTA    1075
P.1/Gamma             RASANLAAIKMSECVLGQSKRVDFCGKGYHLMSFPQSAPHGVVFLHVTYVPAQEKNFTTA    1078
Beta/B.1.351          RASANLAATKMSECVLGQSKRVDFCGKGYHLMSFPQSAPHGVVFLHVTYVPAQEKNFTTA    1075
                      ******** ***************************************************

SARS-CoV-2            PAICHDGKAHFPREGVFVSNGTHWFVTQRNFYEPQIITTDNTFVSGNCDVVIGIVNNTVY    1138
Delta/B.1.617.2       PAICHDGKAHFPREGVFVSNGTHWFVTQRNFYEPQIITTDNTFVSGNCDVVIGIVNNTVY    1138
Omicron/B.1.1.529     PAICHDGKAHFPREGVFVSNGTHWFVTQRNFYEPQIITTDNTFVSGNCDVVIGIVNNTVY    1135
Alpha/B.1.1.7         PAICHDGKAHFPREGVFVSNGTHWFVTQRNFYEPQIITTHNTFVSGNCDVVIGIVNNTVY    1135
P.1/Gamma             PAICHDGKAHFPREGVFVSNGTHWFVTQRNFYEPQIITTDNTFVSGNCDVVIGIVNNTVY    1138
Beta/B.1.351          PAICHDGKAHFPREGVFVSNGTHWFVTQRNFYEPQIITTDNTFVSGNCDVVIGIVNNTVY    1135
                      ***************************************.********************

SARS-CoV-2            DPLQPELDSFKEELDKYFKNHTSPDVDLGDISGINASVVNIQKEIDRLNEVAKNLNESLI    1198
Delta/B.1.617.2       DPLQPELDSFKEELDKYFKNHTSPDVDLGDISGINASVVNIQKEIDRLNEVAKNLNESLI    1198
Omicron/B.1.1.529     DPLQPELDSFKEELDKYFKNHTSPDVDLGDISGINASVVNIQKEIDRLNEVAKNLNESLI    1195
Alpha/B.1.1.7         DPLQPELDSFKEELDKYFKNHTSPDVDLGDISGINASVVNIQKEIDRLNEVAKNLNESLI    1195
P.1/Gamma             DPLQPELDSFKEELDKYFKNHTSPDVDLGDISGINASFVNIQKEIDRLNEVAKNLNESLI    1198
Beta/B.1.351          DPLQPELDSFKEELDKYFKNHTSPDVDLGDISGINASVVNIQKEIDRLNEVAKNLNESLI    1195
                      *************************************.**********************
```

```
SARS-CoV-2              DLQELGKYEQYIKWPWYIWLGFIAGLIAIVMVTIMLCCMTSCCSCLKGCCSCGSCCKFDE      1258
Delta/B.1.617.2         DLQELGKYEQYIKWPWYIWLGFIAGLIAIVMVTIMLCCMTSCCSCLKGCCSCGSCCKFDE      1258
Omicron/B.1.1.529       DLQELGKYEQYIKWPWYIWLGFIAGLIAIVMVTIMLCCMTSCCSCLKGCCSCGSCCKFDE      1255
Alpha/B.1.1.7           DLQELGKYEQYIKWPWYIWLGFIAGLIAIVMVTIMLCCMTSCCSCLKGCCSCGSCCKFDE      1255
P.1/Gamma               DLQELGKYEQYIKWPWYIWLGFIAGLIAIVMVTIMLCCMTSCCSCLKGCCSCGSCCKFDE      1258
Beta/B.1.351            DLQELGKYEQYIKWPWYIWLGFIAGLIAIVMVTIMLCCMTSCCSCLKGCCSCGSCCKFDE      1255
                        ************************************************************

SARS-CoV-2              DDSEPVLKGVKLHYT     1273
Delta/B.1.617.2         DDSEPVLKGVKLHYT     1273
Omicron/B.1.1.529       DDSEPVLKGVKLHYT     1270
Alpha/B.1.1.7           DDSEPVLKGVKLHYT     1270
P.1/Gamma               DDSEPVLKGVKLHYT     1273
Beta/B.1.351            DDSEPVLKGVKLHYT     1270
                        ***************
```

Figure S3. Multiple sequence alignment and construction of the phylogenetic tree of SARS-CoV-2 VOCs. A) Phylogenetic tree of SARS-CoV-2 Spike protein VOCs. **B)** Multiple sequence alignment of the Spike protein VOCs. The domains are colored as: signal peptide (orange); N-terminal domain (green), RBD (red), SD1 (pink) and SD2 (purple), cleavage sites (S1/S2 and S2' are colored in black) the fusion peptide (brown), HR1 (cyan and blue), HR2 (dark green), a transmembrane domain (grey), and cytoplasm domain (not colored) [5,6]. The predominant residues D614G and N501Y are pointed using arrows. The residues of the Spike protein involved in hACE2 interaction are highlighted: E1 region in yellow (corresponds to residues 417, 455-456, 470-490) and E2 region in light blue (made up of residues 444-454 and 493-505) [55]. Multiple sequence alignment and the phylogenetic tree were performed using the ClustalW server [14].

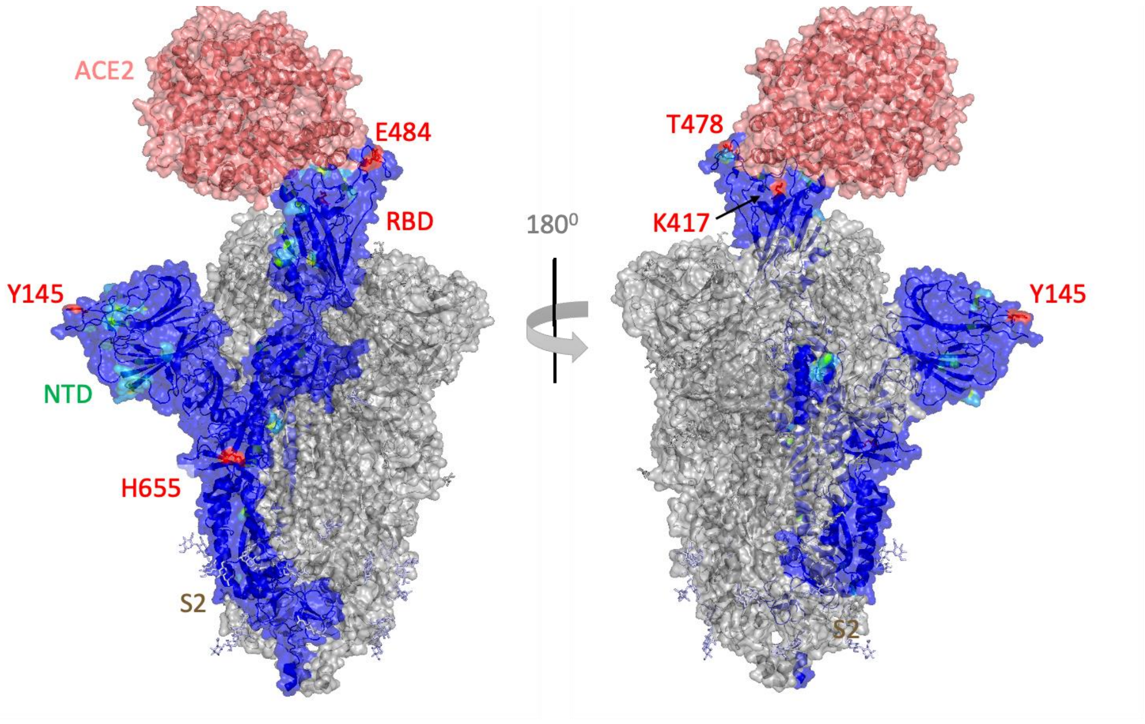

Figure S4. Conservation profile of the Spike protein among VOCs. The surface of the structure of the Spike^{WT} protein in complex with ACE2 (PDB ID: 7DF4 [17]) is colored based on the conservation profile. The ACE2 structure is colored in salmon, and the chains A and C in the trimeric form of Spike protein are colored in grey. The residues Y145, K417, T478, E484 and H655 are colored in red and correspond to the residues in the SARS-CoV-2 VOCs with more variability.

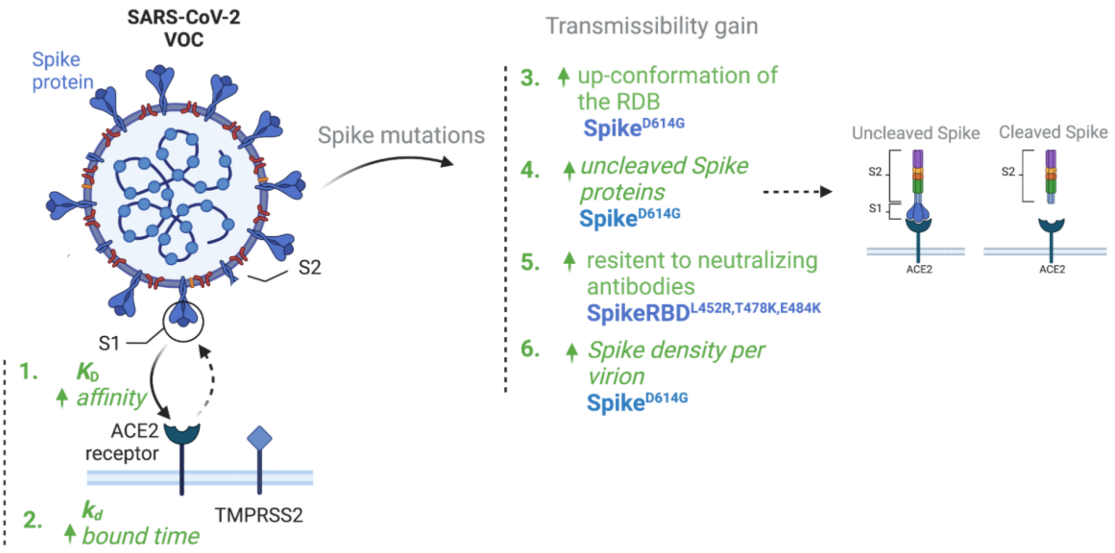

Figure S5: Mutations in the Spike protein of SARS-CoV-2 VOCs result in different mechanisms to evade the immune system, increase the infectiveness, and spread the virus faster in the population. Spike protein of SARS-CoV-2 VOCs increase the hACE2 affinity (K_D) (1); increase the time that the Spike protein is bound to hACE2 (k_d), favoring the process of membrane fusion (2); increase the up conformation of the RBD in the trimeric state of the Spike protein, a conformation that is able to bind to hACE2 (3); increase the amount of the uncleaved form of the Spike protein in virion particles (4); different mutations in the RBD improve resistance to neutralizing antibodies (5); and expand the concentration of Spike proteins per viral particles (6). All mechanisms described contribute to Spike's access to hACE2 and to membrane fusion. In the topic 4 spike cleaved protein refers to the spike protein that is naturally cleaved in the mature viral particle. Mutations in the Spike protein result in greater protection from the proteolysis of the spike protein resulting in an increased amount of complete Spike protein (uncleaved) on the virion surface.

Table S1. Conservation score in the Consurf Server analysis [15,16]. The Spike protein residues with the highest values are listed. High values meaning not conserved residues in the Spike protein among SARS-CoV-2 VOCs.

Spike Residue	Conservation Score
L18	4.5
T19	4.0
P26	4.4
H69	4.1
D80	4.2
T95	4.0
D138	4.2
Y145	7.5
H146	4.3
E156	4.3

R190	4.2
N211	4.3
L212	4.5
V213	4.7
R214	5.0
D215	4.2
G339	4.6
S371	4.2
G339	4.6
S371	4.2
S373	4.0
S375	4.1
K417	8.6
N440	4.1
G446	4.4
L452	4.5
S477	3.9
T478	7.2
E484	7.3
Q493	4.3
G496	4.4
Q498	4.3
N501	4.5
Y505	4.4
T547	4.2
D614	4.5
H655	7.2
A701	4.0

**T716**	**4.0**
**N764**	**4.1**
**D796**	**4.4**
**N856**	**4.1**
**D950**	**4.1**
**Q954**	**4.2**
**N969**	**4.1**
**L981**	**4.5**
**S982**	**3.9**
**T1027**	**4.0**
**D1118**	**4.2**
